# Perception of the threat of War in Israel- implications for future preparedness planning

**DOI:** 10.1186/s13584-015-0026-7

**Published:** 2015-10-01

**Authors:** Moran Bodas, Maya Siman-Tov, Shulamith Kreitler, Kobi Peleg

**Affiliations:** The Department of Disaster Medicine, Sackler Faculty of Medicine, School of Public Health, Tel-Aviv University, P.O. Box 39040, Ramat Aviv, Tel-Aviv 69978 Israel; Sheba Medical Center, Israel National Center for Trauma & Emergency Medicine Research, The Gertner Institute for Epidemiology and Health Policy Research, Tel Hashomer, Ramat-Gan Israel; Psychoncology Research Center, Sheba Medical Center, Gordon Faculty of Social Sciences, School of Psychological Sciences, Tel-Aviv University, Tel Hashomer, Ramat-Gan Israel

**Keywords:** Emergency, Preparedness, War, Perception of threat, Israel

## Abstract

**Background:**

It has been recently reported that the preparedness of the Israeli public to a war scenario is mediocre. These findings suggest a need to study the psychosocial mechanisms behind individual motivation to engage in preparedness behavior. One component of these mechanisms is the perception of threat. The purpose of this study is to portray the perception of the threat of war by the Israeli public and to deduce possible implications for resilience-promoting policies.

**Methods:**

Portions of the data accumulated in a telephone-based random sampling of 503 households (representing the Israeli population) performed in October 2013 were utilized to examine the perception of the threat of war by Israelis. The questionnaire was used to examine the level of household preparedness, as well as attitudes toward perception of threat, preparedness responsibility, willingness to search for information, and sense of preparedness. Statistical analysis was performed to determine the correlations between different components of threat perception, and to evaluate the preparedness promoting features of specific perception factors.

**Results:**

The data suggest that the perception of threat is influenced by different socio-demographic factors. In particular, age, religion and education seem to play an important role in the perception of threat. Compared to data collected almost a decade ago, the likelihood perception and threat intrusiveness rates were significantly reduced. The regression analysis suggests that perception of the severity of the impact on a family’s routine and willingness to search for information, two known preparedness promoting factors, can be predicted by various socio-demographic and threat perception components.

**Conclusion:**

The data suggest that the Israeli public, post the Second Lebanon War (2006) and the Gaza conflicts of 2009 and 2012, perceives the probabilities of war and being affected by it as diminished. The Israeli public demonstrates what can be considered as the unique characteristics of a war-victimized population. Implications for a future resilience-promoting policy were discussed.

## Background

Each year, emergency and disaster situations claim a heavy toll in human lives and economic loss [1]. The literature provides ample support for the claim that civilian populations, that are more prepared for emergencies also react better in the face of crisis and are less vulnerable to its adverse effects [2, 3]. There is also evidence to support the viewing of the preparedness at the family level as a building block for communal resilience to emergencies [4–10]. In particular, this notion was found to be applicable to the Israeli public [11]. It is generally accepted that households engaging in emergency preparedness activities are potentially more resilient than others when confronted with an actual crisis, and therefore, are expected to require less medical care in the aftermath of the crisis [7, 8].

In an effort to explore this aspect in Israel, we reported in a separate publication that the preparedness of the Israeli public to a war scenario is mediocre [12]. The findings suggested that half of the Israeli population has complied with 50 % or less of the civil defense recommendations for household preparedness to this threat. The results also suggested that in contrast to other populations, the Israeli public demonstrates little correlation between perception components and preparedness behavior. These findings led the proposal for an additional study of the perception of threat and its association to preparedness behavior, in order to better understand preparedness behavior. More specifically, in order to promote civilian resilience to emergencies, it is imperative that the psychosocial mechanisms behind individual motivation to engage in preparedness behavior be studied. Looking into the perception of threat could constitute a preliminary step in this direction.

A review of the literature in the field of emergency preparedness has identified several components of the perception of threat that were found to correlate with a household’s level of preparedness [13–19]. Among these are: (1) the perception of the likelihood of the threat occurring in the near future; (2) the perception of the likelihood of personal harm (also known as threat intrusiveness); and (3) the perception of the severity of the threat to different elements of the social fabric. In addition, the perception of the responsibility to engage in preparedness behavior, specifically the tendency to assume personal responsibility, correlated with actual preparedness behavior [20–26].

Since these components were part of the questionnaire that was utilized for the national preparedness survey presented in Bodas et al. (2015) [12], the data accumulated from that survey was used to study the perception of the threat of war by the Israeli public in this study.

## Methods

### Sample & setting

A random digital-dial (RDD) sample of telephone numbers drawn from the population registry of all area codes in Israel was conducted by a polling company between October 20–25, 2013. The telephone survey resulted in a sample of 503 households representing the population of the State of Israel (Table 1). The response rate was 23 %, and it is considered commonplace in telephone-based surveys [27]. The sampling took place amid rising tensions on the Syrian-Israeli border following the use of chemical weapons by the Syrian regime on the Syrian population, which intensified the aggressive posturing in the region. These rising tensions in turn led to a surge in the demand for gas masks in Israel and increased the level of awareness of the possibility of war among the Israeli public.

**Table 1:** Socio-demographic distribution of studied sample (N = 503)

Variable	*n* (%)
Gender	
Female	260 (51.7 %)
Male	243 (48.3 %)
Age	
18-30	137 (27.2 %)
31-50	192 (38.2 %)
51-70	133 (26.4 %)
71-99	40 (7.9 %)
Missing	1 (<1.0 %)
Marital Status	
Married	319 (63.4 %)
Other (single, divorced, widowed)	184 (36.6 %)
Birth place and immigration	
Israel	335 (66.6 %)
Veteran immigrant (prior to 1991)	104 (20.7 %)
New immigrant (1991 onward)	64 (12.7 %)
Place of residence^a^	
North or south	230 (45.8 %)
Other (Center, Negev, greater Jerusalem, Judea & Samaria)	272 (54.1 %)
Missing	1 (<1.0 %)
Religion	
Jews	370 (73.5 %)
Muslims	85 (16.9 %)
Others	48 (9.6 %)
Affiliation to religion	
Secular	272 (54.1 %)
Traditional	135 (26.8 %)
Religious	69 (13.7 %)
Ultra-orthodox	27 (5.4 %)
No. of children under 18 y/o	
0 (None)	263 (52.3 %)
1-3	200 (39.7 %)
≥4	40 (8.0 %)
Education	
< K-12	126 (25.0 %)
High-School diploma	129 (25.7 %)
Vocational education	54 (10.7 %)
Academic education	194 (38.6 %)
Income	
Less than average	119 (23.7 %)
Average	125 (24.8 %)
More than average	171 (34.0 %)
Missing	88 (17.5 %)

^a^ For the purpose of this study, the geographical distribution of participants was determined according to regions of past conflicts. The northern and southern areas referred to in this research were those affected by the Second Lebanon War (2006), and “Cast Lead” (2009) and “Pillars of Defense” (2011) operations

### Tools

A questionnaire examining the components of threat perception and the preparedness of responders was developed based on an extensive literature review. The final version of the questionnaire was attained following preliminary studies that examined the clarity of the different items to different participants and a pilot study involving 226 people.

This current study deals with the first set of items included in this questionnaire. The perception variables measured and assessed were: (a) likelihood of war taking place in the next year or five years- two items; (b) the likelihood of being personally affected by war, i.e., injury or death- one item; (c) threat severity to different elements of the societal fabric, i.e., general society, community, family’s routine, family property and family members (injury or death)- five items (Cronbach’s Alpha: 0.854); and (d) responsibility for engaging in preparedness behavior by assigning responsibility to different factors e.g., state, military, local authority, community, family and self- six items (Cronbach’s Alpha: 0.792). All of these were measured using multiple-choice items, in which a Likert-based scale was applied. Items requiring a more decisive answer by responders were assigned an even number of answers, thus eliminating a mid-scale option, which is known to distort the response distribution in some cases [28]. In odd scales, the optional answers were rated on a scale of 1 (very low) to 5 (very high), while in even scales, the middle answer was removed.

The willingness to search for information index was calculated as the mean score of an agreement with four statements: (Cronbach alpha: 0.629) (a) When I read or hear the news, I find interest mostly in news concerning a possible war in Israel in the near future; (b) I often look for preparedness information on my own initiative; (c) If I were to be invited to a preparedness seminar I would attend; (d) I am willing to accept a Home Front Command representative at my home to learn more about emergency preparedness.

### Statistical Analysis

The statistical analysis of the results was performed using SPSS Version 22. The analysis included both descriptive and analytical methods, and the statistical tests were chosen according to the distribution of variables. The correlations between categorical variables were examined using a Chi-Square test, associations between categorical and continuous variables were examined with *T*-test for independent samples, and the Spearman Correlation Test was used to examine correlations between continuous variables.

In addition, regression analyses were employed to predict the perception of threat and the willingness to search for information. The first was examined through a multi-variant, binary-logistic regression analysis to determine the odds-ratios of perception of threat components according to socio-demographic variables that were found to correlate with those components. This regression analysis was first carried out unadjusted for each independent variable correlating with the specific perception component, and then, adjusted for all variables. In this manner, the validity of the results could be assessed.

The second regression analysis was implemented using a multi-variant linear regression analysis to predict the willingness of participants to actively look for information on emergency preparedness. The regression was performed in Stepwise mode with two blocks: the first step included demographic variables, and the second step included components of the perception of threat. Only variables that were found to be associated with the dependent variable, following the negation of multiple co-linearity, were introduced into the regression analysis.

In all statistical analyses performed, a p-value of 0.05 or less was determined as statistically significant.

## Results

When asked to assess the likelihood of war irrupting in Israel, 22 % and 40 % of the sample predicted “high” or “very high” chances of such an occurrence happening within the next one or five years, respectively. Analysis carried out to establish the differences in the perception of likelihood between people of different socio-demographic background revealed that Jews and people under 40 years of age were more than twice likely to perceive the chances of war occurring within the next year as higher, compared to non-Jews and people over 40 years old. The same trend was observed for the five-year time frame in which differences were also observed between people with an academic education and average to low incomes, compared to non-academics and high earning households, but these were not significant in the multivariate adjusted analysis (Table 2).

**Table 2:** Adjusted and unadjusted odds-ratio of threat perception components according to correlated socio-demographic variables (*N* = 503)

Threat perception component	Variable^a^	Unadjusted		Adjusted	
		OR	95 % CI	OR	95 % CI
Likelihood perception (1 year)	Religion	2.08	(1.375, 3.143)^++^	2.16	(1.415, 3.286)^+++^
	Age	2.03	(1.418, 2.900)^+++^	2.10	(1.456, 3.017)^+++^
Likelihood perception (5 year)	Religion	3.14	(2.063, 4.768)^+++^	3.29	(2.050, 5.297)^+++^
	Age	1.70	(1.156, 2.490)^++^	1.72	(1.099, 2.697)^+^
	Education	1.78	(1.193, 2.668)^++^	1.61	(0.992, 2.606)
	Income	0.59	(0.385, 0.900)^+^	0.64	(0.409, 1.014)
Severity perception (to society)	Gender	0.63	(0.413, 0.956)^+^	0.62	(0.408, 0.963)^+^
	Age	1.71	(1.113, 2.618)^+^	1.73	(1.120, 2.680)^+^
	Place of birth	2.81	(1.606, 4.904)^+++^	2.87	(1.630, 5.073)^+++^
Severity perception (to family routine)	Education	1.62	(1.034, 2.528) ^+^	NR	
Responsibility perception (Self)	Place of residence	0.57	(0.402, 0.819)^++^	0.59	(0.410, 0.837)^++^
	Children under 18	1.44	(1.010, 2.047)^+^	1.40	(0.982, 2.005)

^a^Only variables found to correlate with the respective dependent variable are shown. The categorization of the independent variables are as follows: Religion: Jews = 1, other = 0; Age: under 40 = 1, over & including 40 = 0; Education: academic = 1, non-academics = 0; Income: more than average = 1, average or low = 0; Place of residence: north or south = 1, other = 0; Gender: male = 1, female = 0; Place of birth: veteran immigrant or native Israeli = 1, new immigrant = 0; Children under 18: yes = 1, no = 0. + *p* < 0.05 ++ *p* < 0.01 +++ *p* < 0.001

When asked to estimate the likelihood of personal harm (i.e., injury or death) as a direct result of a war outbreak, the majority (55 %) of participants assessed it as low, compared to 33 % who assessed this likelihood as high. Twelve percent refused to answer this particular question - the highest rate of refusal for a specific item in this study. Threat intrusiveness was reported more by residents of areas where conflicts had occurred previously, i.e., the north and south of Israel (2.31 ± 1.01), when compared with residents of other regions (2.11 ± 0.92) according to independent *T*-test (t = −2.12, df = 439, p = .035). However, this finding was not statistically significant when examined in the binary-logistic regression (Table 2).

The results of the severity perception indicate a tendency to gravely predict the potential outcomes of a war occurring in Israel in the near future; 76.4 % of the participants predicted high severity of the impact to the routine of life of the entire population, 69.2 % to the routine of the local community, 75.8 % to the routine of their families, 59.7 % to their personal property, and 56.4 % to themselves and their family members (i.e., injury or death). It is interesting to note that the percentage of refusal to answer a specific item increased, as the items progressed closer to the participant, ranging from a 2.4 % refusal rate for the first item to 7.4 % for the last item. As revealed by the independent samples *T*-test (data not shown), when looking into the influence of socio-demographic variables on the perception of severity, it was observed that women, people aged under 40 and native-born Israelis perceive the severity of war as higher, in comparison to men, people over 40 years old and immigrants, respectively. The regression analysis performed to explore these results found that men were about 60 % less prone to perceive the threat more severely than women, and that being a native-born Israeli or a veteran immigrant is predicted to increase almost three-times the severity perception of the threat compared to a new immigrant (Table 2).

To complete the set of severity perception results, participants ranked the war scenario in comparison to other emergencies. The results suggest that the war scenario is perceived to be more severe than a major forest fire (24.6 % of opinionated participants replied the latter is worse), more severe than an economic crisis (35.8 %) and a terror attack (42.8 %), and less severe than a major earthquake (73.7 %), a nuclear attack (87.9 %) and a chemical attack (89.3 %).

When asked to assign different organizations with the responsibility of engaging in preparedness efforts, 54.5 % of participants attributed high or very high responsibility to themselves, 57.5 % to their families and local communities (each), 76.5 % to their local authority, 89.3 % to the military and civil defense authorities, and 89.9 % to the government.

According to independent samples *T*-test (data not shown), residents living outside of previous conflict areas (i.e., Center, Negev, Greater Jerusalem, and Judea & Samaria districts) and parents of children under 18 years of age were more likely to assume a personal responsibility for preparedness than residents of the north and south and participants without children under 18 years of age. These findings were also partially supported by the regression analysis, with the exception that having children under 18 years old was not found to be statistically significant in the multivariate adjusted analysis (Table 2).

Finally, correlations were observed between most components of the perception of threat, as well as between the perception of responsibility and the severity of the threat (Table 3).

**Table 3:**
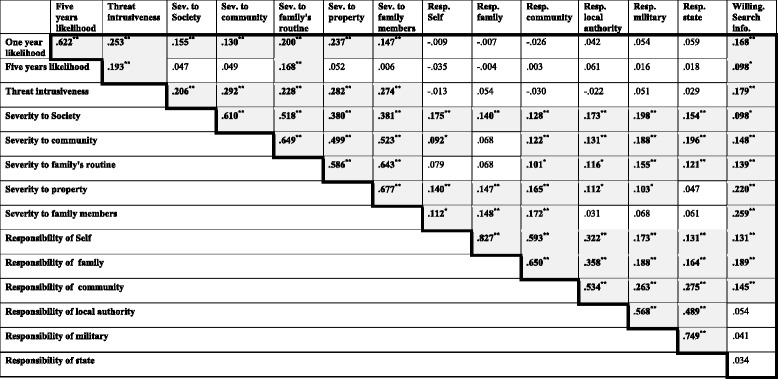
Spearman Correlations of threat perception components of war in Israel (*N* = 503)

*Sev* Severity; *Resp* Responsibility; *Willing. search info*. Willingness to search for information. Statistically significant results are highlight

^*^ Correlation is significant at the 0.05 level (2-tailed)

^**^Correlation is significant at the 0.01 level (2-tailed)

In addition, it is interesting to explore the predictors of the perceived severity of impact on a family’s routine and willingness to search for information, factors that were reported to correlate with preparedness behavior in a previous publication [12]. The regression analysis suggested that the model behaves differently for new immigrants and native born or veteran immigrants. A stratified examination of the data indicated that while the severity perception among new immigrants cannot be effectively predicted by other variables, it could be predicted among native Israelis by education and income levels (in an adjusted analysis). Holders of an academic degree were three times more likely to perceive the greater severity of the threat than participants with a non-academic education (OR = 3.33 95%CI 1.731, 6.405 *p* < .001). Similarly, higher earning individuals were almost twice as likely to perceive greater severity than average to low income earning individuals (OR = 1.824 95%CI 1.060, 3.137 *p* = .03).

The regression analysis performed to predict the willingness to actively search for information revealed that 22.4 % of the dependent variable can be predicted by religion, affiliation to religion, education, age, perception of the threat likelihood (within the next year) and perception of threat severity to self and family members. The predominant variable in predicting willingness to search for information was the latter (β = 0.309) (Table 4).

**Table 4:** Results of final step of linear regression analysis to predict willingness to search for information

Variable	Categories	β	*p*-value	R^2^
Religion	0 - other	-.263	.000	.224
	1 - Jewish			
Affiliation to religion	0 - religious	-.135	.006	
	1 - secular			
Education	0 - non-academic	-.161	.001	
	1 - academic			
Age	Cont.	.145	.003	
Severity to self and family members	Cont.	.309	.000	
One year likelihood	Cont.	.147	.003	

Regression analysis performed in stepwise mode with two block. The variables entered into the analysis were: First block - gender, age, religion, affiliation to religion, education and income. Second block – one year likelihood, five years likelihood, threat intrusiveness, severity to society, severity to community, severity perception to family’s routine, severity to property, and severity to self and family members

## Discussion

The purpose of this study was to explore the perception of the threat of war by the Israeli public. The motivation behind the research arises from evidence provided by the literature for association between the perception of threat and preparedness behavior [13–19]. However, this does not seem to be the case with the Israeli population, at least not for the war scenario. In a recent publication based on the same database presented in this paper, it was demonstrated that the Israeli public did not exhibit the correlation reported in the literature between perception of threat and preparedness, but for one exception concerning the perceived severity of impact on the family’s routine [12]. Given this seeming disparity between the threat perception components and the preparedness behavior, one could argue that there is no reason to further explore the perception of threat by the Israeli public because no meaningful conclusions can be drawn for policymaking. Yet, as the findings of this study suggest, there is much to be learned from the analysis of the perception of threat by the Israeli public concerning better approaches to promote public resilience.

It is interesting to note that the Israeli public demonstrates unique patterns of threat perception, when compared to those reported for other populations. On the one hand, Israelis tend to estimate the likelihood of war in the near future as low. In actuality, this assessment turned to be false with the eruption of the July 2014 operation in Gaza, less than a year following our telephone-based survey. On the other hand, Israelis tend to estimate the severity of the threat as high, with more than half of the sample anticipating severe outcomes for themselves, their family members and their properties. For both variables, i.e., the perception of likelihood and the perception of severity, younger people tend to have a more pessimistic view of the threat. Nevertheless, explaining this phenomenon by “young and afraid” simply cannot cut it because the data also suggest a complimentary trend, according to which the more experienced a person is with the threat, the more he or she perceives it as a threat. This is demonstrated through the higher rates of the perception of severity among native-born Israelis, and the higher threat intrusiveness among residents of regions affected by prior conflicts; both represent groups that endured the threat for longer extents than their counterparts did.

The above hypothesis regarding the unique characteristics of the Israeli public’s perception of threat is supported by findings reported by Lahad, Shcham & Shcham (2009) [29], who have examined the Jewish and Arab population in northern Israel following the Second Lebanon War. The authors concluded that communities enduring persisting hardship such as war and terrorism, have less faith in authorities’ capabilities to support preparedness and are less likely to anticipate retrieval of normalcy to their everyday lives. Mistrust in authorities was already reported as counterproductive to individual preparedness behavior [30].

Perhaps, one of the most important findings of this research emerges from the changes observed in the patterns of threat perception by the Israeli public in recent years. In 2005, Kirschenbaum noted that 66 % of his then studied sample of the Israeli public reported perceiving a medium to high chance of war occurring in Israel in the “near future.” [17] However, less than a decade later, we observed a reduction in the likelihood perception with only 49 % of the current sample reporting similar likelihood rates (in adjusted scales). According to the Chi-square test (χ ^2^ = 48.081, df = 2, *p* < .001), this difference is statistically significant. In line with the Chi-square test (χ ^2^ = 5.95, df = 1, *p* = .015), threat intrusiveness rates reported in the current study (33 %) were significantly lower than those reported by Kirchenbaum in 2005 (44 %). The data suggest that the Israeli public, post the Second Lebanon War (2006) and the Gaza conflicts of 2009 and 2012 perceives the probabilities of war and being affected by it as diminished. Attempting to generalize this conclusion, these results suggest that kept under constant reminder of a threat, a given population might develop apathy or indifference toward it.

We suggest that the reduction in threat intrusiveness can be explained as the result of habituation to armed conflicts, a process in which the Israeli public is desensitized to the threat by observing a *relatively* small toll on Israeli lives. A similar notion was proposed by Dov Waxman (2011) for terrorism [31]. This phenomenon is backed in numbers. According to official data [32–34]: (a) during the 33 days of the Second Lebanon War (2006), 41 civilians died and 2000 were injured. Approximately 4000 rockets were fired at the northern part of the country, thus, generating a death rate of one person per 100 rockets; (b) during the 22 days of operation “Cast Lead” (2009) in Gaza, three civilians were killed and 183 were injured as a result of the 536 rockets fired during this conflict - a death rate of 0.6 persons per 100 rockets; (c) in the eight days of operation “Pillar of Defense” (2012) in Gaza, 4 civilians were killed and 241 injured as a result of the 1264 rockets not intercepted by the “Iron Dome” missile defense system. This constitutes a death rate of 0.3 persons per 100 rockets. A similar trend can be observed in the rates of injured persons. Although not relevant for the data reported in this study performed in 2013, the trend continues for the 2014 Gaza conflict, in which the death rate dropped to 0.14 persons per 100 rockets not intercepted.

The data provided above suggest that the Israeli public, possibly reassured by a technological advancement in the defense arena (e.g., the “Iron Dome” missile defense system), have grown accustomed to the threat of high-trajectory weapons, and perceive them less-and-less as a personal risk. Yet, this should also be considered for its backfiring potential, since the same apathy can lead people to place themselves in harm’s way instead of following lifesaving instructions. This phenomenon was already observed during the 2009, 2012, and 2014 Gaza conflicts, in which civilians were either killed or injured because of their disregard or non-adherence to emergency behavior instructions issued by the civil defense authority.

While the reduction in threat intrusiveness since 2005 might be explained by the habituation effect, it is much more difficult to provide an explanation for the parallel reduction in the perception of the likelihood of the threat. This issue is further complicated in light of the tendency to expect the worst out of the war scenario, as demonstrated in the severity perception data. The findings here seem to be conflicting. On the one hand, the majority of Israelis do not foresee a looming war in the near future; however, they also hold a grave perception of the outcomes of such war if it indeed occurred. In order to explain the reduced rates of likelihood perception, one should first explain the findings of the severity perception.

This study suggests that the Israeli public is anticipating severe outcomes of a war taking place in Israel in all layers of its societal fabric, including the impact on the family’s routine. Despite enduring this threat for decades, the notion of war remains intimidating to most Israelis. It is therefore not surprising that responders tend to alienate themselves from the risk. Responders in our survey increasingly refused to answer items as they became more specific to their personal well-being. Potentially, this could be explained as a basic mechanism of denial, and could account for much of the findings presented in this paper: a perception of a severe threat may lead to denial-based coping mechanisms that are exhibited in a reduced perception of likelihood. This serves to further illustrate the difficulty in motivating the public to engage in preparedness behavior. In this context, it is also interesting to note that people residing in areas affected by armed conflict up to 2013, i.e., the north or south of Israel, are also less keen on assuming personal responsibility for preparedness behavior. This finding suits the suggested explanation that repeated experience with the threat could be counterproductive in promoting individual motivation for an engagement in preparedness behavior.

The findings of this study suggest that the association reported in the literature between the perception of threat and preparedness behavior is probably mediated by other factors, which are yet to be fully identified. A similar notion was proposed by Rüstemli and Karanci (2010), who examined the preparedness behavior among earthquake-victimized population in Turkey [35]. The authors conclude there:

*These results suggest that protective behavior in victimized populations is determined essentially by fear and belief in personal control rather than severity of prior experience and cognition related to the perceived severity and occurrence of future hazards.* (p. 99)

### Implications for policymaking

In a separate publication [12] based on the same database examined in this paper, we reported correlates of preparedness behavior in the Israeli public. It was reported that 15.4 % of the variance in preparedness behavior, i.e., the public’s compliance rate with the civil defense recommendations, can be explained by place of birth, place of residence, sense of preparedness, willingness to search for information, and the perceived severity of impact on a family’s routine. Out of these five predictors, only the last two can be considered as targets for potential intervention policies.

In this current study, it is demonstrated that the perceived severity of impact on a family’s routine could be predicted through levels of income and education (for native-born Israelis). However, it should be noted that our previous study reported a negative correlation between severity perception and preparedness behavior [12]. Since we cannot and do not wish to propose to promote public resilience by increasing the severity perception at the expense of reducing literacy rates or income, we cannot offer a meaningful recommendation on this aspect.

Nevertheless, there is much to be learned from examining the data around the willingness to search for information. Other publications support the beneficial attributes of willingness to search for information [15, 26, 36, 37]. More recently, a national survey performed by the U.S. Federal Emergency Management Authority (FEMA) in 2012 found that information aware respondents were more likely than unaware respondents to report that they had updated their supplies and had a household emergency plan [38]. In this current study, the regression analysis suggests that this preparedness-promoting factor can be predicted by several socio-demographic variables, but also by perceptions of likelihood and severity. In fact, severity perception to oneself and family members was the most predictive factor of willingness to search for information (β = 0.309). In the face of it, this seems to contradict the previously reported negative effect observed for severity perception on preparedness behavior [12], because it suggests that having a graver perception of threat outcomes can lead to information seeking, which is predicted to increase engagement in preparedness behavior. Since the negative correlation reported between severity perception and preparedness [12] did not point out which is the cause and which is the effect, the latest findings reported in this study suggest that increased preparedness leads to a decrease in severity perception, almost certainly due to a heightened sense of preparedness. A full picture can now be proposed – increased severity perception of the threat to oneself and family members promotes information seeking, which is expected to result in actual preparedness behavior. Once this behavior is performed, the individual appraisal of increased familial preparedness leads to a sense of preparedness that decreases the perceived severity of impact on a family’s routine. These findings suggest that a possible target for risk communication strategies should be promoting individual assessment of personal and kin risk prior to and following preparedness behavior.

Lastly, investing in the promotion of public resilience to emergency can translate into reduced demands for healthcare in the aftermath of a crisis. This is probably true for the provision of both medical healthcare and mental healthcare. Drawing support from the literature in the field [2, 3, 7, 8], we conclude that a more resilient Israeli public will be able to better cope in the face of adversity. Policy makers should therefore regard the promotion of resilience as an additional means of promoting public health through preventive measures.

## Conclusions

The findings of this study suggest that the Israeli public demonstrates unique patterns of perception of the threat of war. Israelis underestimate the likelihood of war, while at the same time overestimate its adverse outcomes. An analysis of these perception patterns, in particular, in comparison to the data provided on the Israeli public from less than a decade ago suggests a possible combined mechanism of denial and habituation to the threat. The findings of this study illustrate the difficult playground set for preparedness promoters such as civil defense authorities.

Since populations demonstrating higher resilience are better fitted to cope with the adverse effects of crisis, policy makers striving to promote public health should also promote resilience. Nonetheless, a population is only as resilient as its members are. It is therefore imperative to study the psychosocial mechanisms behind individual motivation to engage in preparedness behavior in order to better understand how to promote public health in this aspect. Once these mechanisms are deciphered, a proper strategy for risk communication and public engagement can be generated.
